# Three Fatal Gestational Psittacosis Cases Caused by *Chlamydia psittaci* Strains Belonging to Closely Related Lineages, Japan

**DOI:** 10.3201/eid3205.252008

**Published:** 2026-05

**Authors:** Atsuko Nishino, Yukiko Nakura, Yukiko Sassa-O’Brien, Momoko Soeda, Hirokazu Sugii, Kanako Shimizu, Shiro Miura, Yumiko Sato, Michinobu Yoshimura, Michiko Kodama, Itaru Yanagihara

**Affiliations:** Research Institute, Osaka Women’s and Children’s Hospital, Osaka, Japan (A. Nishino, Y. Nakura, M. Yoshimura, I. Yanagihara); The University of Osaka, Osaka (A. Nishino, M. Kodama, I. Yanagihara); Tokyo University of Agriculture and Technology, Tokyo, Japan (Y. Sassa-O’Brien); NHO Nagasaki Medical Center, Nagasaki, Japan (M. Soeda, S. Miura); NHO Iwakuni Clinical Center, Yamaguchi, Japan (H. Sugii, Y. Sato); Tannan Health Welfare Center, Fukui, Japan (K. Shimizu); Maizuru Kyosai Hospital, Kyoto, Japan (K. Shimizu)

**Keywords:** *Chlamydia psittaci*, bacteria, zoonoses, gestational psittacosis, multilocus sequence typing, Japan

## Abstract

Gestational psittacosis is a rare infectious disease caused by *Chlamydia psittaci* that causes high maternal and fetal mortality rates. In Japan, gestational psittacosis has been reported in 7 patients, including 4 maternal deaths without antemortem diagnosis. We molecularly diagnosed *C. psittaci* infection postmortem in 3 patients treated during 2017–2024. We extracted DNA from formalin-fixed paraffin-embedded placenta, lung, and spleen tissues. Analysis of multilocus sequence typing indicated sequence type (ST) 269 in 1 patient and ST335 in 2; all 3 were closely related lineages that have not been previously reported in Japan or in animals. However, the *ompA* gene showed distinct clusters in the phylogenetic analysis. Quantitative PCR and immunostaining revealed higher amounts of *C. psittaci* detected in placenta than in lung or spleen, suggesting that proliferation of *C. psittaci* in the placenta might cause severe symptoms. ST335/ST269 lineage could be highly virulent strains for pregnant women.

Gestational psittacosis is a rare infectious disease caused by *Chlamydia psittaci* and is associated with high maternal and fetal mortality rates (*1*). *C. psittaci* is an obligate intracellular gram-negative bacterium that has been reported to cause 1%–2% of community-acquired pneumonia among hospitalized patients annually (*2*). The mortality rate for psittacosis when including nonpregnant periods is <1% with proper treatment. During pregnancy, the maternal immune system accepts the semiallograft fetus by controlling T helper (Th) 1/Th2 balance, T regulatory (Treg) cell activity, and Th9 to achieve immunologic tolerance (*3*). However, that immunotolerant status might reduce the protective nature against intracellular invasive bacteria, such as *Listeria monocytogenes*, *Coxiella burnetii*, and *Chlamydia* spp., resulting in adverse outcomes (*4*). Although fetal cases of gestational psittacosis have been reported less frequently than fetal pneumonia cases, gestational psittacosis poses a threat to life; rates of intrauterine fetal mortality (82.6%, 19/23) and maternal mortality (8.7%, 2/23) are high (*5*). Given that the maternal mortality rate is reported to be ≈9 times higher than the overall case fatality rate of psittacosis, rapid diagnosis and emergency medical care are necessary. 

Psittacosis in humans is a notifiable disease in Japan; 5–11 cases were reported annually in recent years (*6*). Birds, particularly species in the families Cacatuidae and Columbidae, are considered the primary source of infection (*7*). Among the reported cases of human psittacosis in Japan during 2007–2016, the suspected source of infection was identified as birds in 79% of cases and unknown or unreported exposure histories in the remaining 21% of cases; the identified birds were from the order Psittaciformes (parakeets/parrots) in 53%, doves or pigeons in 35%, and other avian species in 12% of cases (*6*). In Japan, the recent prevalence of *C. psittaci* is <1% among pet birds but remains unclear among wild birds, although detection in pigeons and feral parrots has been reported (*6*,*8*–*10*). Reports on infections from wild birds, particularly outbreaks in Europe during 2023–2024, further illustrate that wild birds can serve as sources of infection (*11*–*13*). Although birds remain the primary source of human psittacosis, transmission between humans or through other mammals, such as horses and Siberian elk (*Alces alces cameloides*), have also been reported (*14*–*16*). This diversity of potential infection routes complicates epidemiologic investigations, and the wide range of hosts has made identifying the infection route more complicated.

In Japan, we have diagnosed 3 fatal gestational psittacosis cases among maternal deaths of unknown etiology during 2017–2024. In this study, we aimed to obtain epidemiologic information on those 3 cases by phylogenetic analysis of chlamydial marker *ompA* and by performing multilocus sequence typing (MLST), a valuable tool for estimating the source of infection with bacteria of the order Chlamydiales. In addition, we evaluated the distribution of *C. psittaci* in placentas, maternal spleens, and maternal lungs using quantitative real-time PCR (qPCR) analyses and immunofluorescent staining to investigate the pathogenesis of gestational psittacosis.

## Materials and Methods

### Samples and DNA Extraction

In cooperation with the Japan Maternal Death Exploratory Committee of the Japan Association of Obstetricians and Gynecologists, we evaluated 3 cases (FO-01 [*17*,*18*], YO-02 [*19*,*20*], and NO-03) of maternal and fetal death of unknown etiology for which autopsies were performed at different locations within Japan during 2017–2024. Formalin-fixed paraffin-embedded (FFPE) tissue samples were sent to our laboratory, and we extracted DNA from the placenta, maternal lungs, and maternal spleen using Maxwell RSC DNA FFPE Kit (Promega, https://www.promega.com) according to the manufacturer’s instructions.

### Molecular Diagnosis of Gestational Psittacosis

We performed PCR to detect chlamydial ribosomal DNA. For initial screening, we performed real-time PCR using the primer pair CPSI_F/CPSI_R, which amplifies the region spanning the 3′-region to the intergenic spacer region of 16S-23S rRNA gene, specific to *C. psittaci* and *C. abortus* (*21*). We prepared a 10-μL reaction mixture containing 5 μL of PowerUp SYBR Green Master Mix (Thermo Fisher Scientific, https://www.thermofisher.com), 0.5 μM of each primer, 3 μL of double-distilled water, and 1 μL of DNA template. The cycle conditions were 40 cycles at 95°C for 15 seconds, then annealing at 55°C for 15 seconds and 72°C for 1 minute. Next, we performed 16S rRNA gene PCR under the same conditions using the primers C.p.16S 45F/C.p.16S 320R and C.p.16S 1172F/C.p.16S 1370R to distinguish among *C. psittaci*, *C. abortus*, and *C. buteonis*. We used the primer pair C.p.16S 45F/C.p.16S 320R to amplify 276 bp 5′-region of 16S rRNA gene and used the primer pair C.p.16S 1172F/C.p.16S 1370R to amplify the 199-bp 3′ region of 16S rRNA gene. We retrieved 16S rRNA gene sequences of *C. psittaci*, *C. abortus*, and *C. buteonis* from the National Center for Biotechnology Information RefSeq database (https://www.ncbi.nlm.nih.gov/refseq). The alignments revealed 5 nucleotide polymorphisms among *C. psittaci*, *C. abortus*, and *C. buteonis*. Among the 5 nucleotide differences identified in the 16S rRNA gene, we selected the regions encompassing 4 of those polymorphic sites as the target for PCR amplification. We purified the PCR amplicons using NucleoSpin Gel and PCR Clean-up (MACHEREY-NAGEL, https://www.mn-net.com) after gel electrophoresis; subjected them to Sanger sequencing using Big Dye Terminator 3.1 kit on an SeqStudio Genetic Analyzer (Thermo Fisher Scientific); and compared them with reference sequences by using BLASTn (https://blast.ncbi.nlm.nih.gov). 

### Multilocus Sequence Typing

We performed PCR of 7 housekeeping genes (*enoA*, *fumC*, *gatA*, *gidA*, *hemN*, *hflX*, and *oppA_3*) using DNA extracted from the placenta, as previously described, and the reported primer sets (Table 1). The gene *hemN* could not be amplified with the previously reported primer sets YPhemN1 and YPhemN2; therefore, we prepared new primers designated as hemN-F2 and hemN-R2. For each sample, we prepared a 15-μL reaction mix by combining 1 μL of sample template, 0.075 μL of TaKaRa Ex Taq, 1.5 μL of 10x Ex Taq Buffer (Mg^2+^ plus), 1.2 μL of deoxyribonucleotide triphosphate mixture (TaKaRa Bio, https://www.takarabio.com), and 1.0 μL of each primer (10-µM stock). The cycle conditions were 35 cycles at 98°C for 10 seconds, 55°C for 30 seconds, and 72°C for 45 seconds. Subsequently, we purified and analyzed the PCR products as described previously. We concatenated the MLST alleles and determined the sequence type (ST) using the PubMLST Chlamydiales database (https://pubmlst.org/chlamydiales). We visualized MLST phylogenetic relationships among *C. psittaci* using GrapeTree, an interactive tool in EnteroBase (https://enterobase.warwick.ac.uk) for visualizing phylogenetic trees. The tool reconstructs and displays complex minimum spanning trees and integrates detailed metadata to enable epidemiologic interpretation (*24*).

**Table 1:** Primers and probes used for diagnosing *Chlamydia psittaci* and MLST in study of 3 fatal gestational psittacosis cases caused by *C. psittaci* strains belonging to closely related MLST lineages, Japan, 2017–2024*

Method	Target gene	Primer or probe	Sequence, 5′→3′	Amplicon size, bp	Reference
Real-time PCR	16S–23S rRNA operon	CPSI_F	AAGGAGAGAGGCGCCCAA	133	(*21*)
		CPSI_R	CAACCTAGTCAAACCGTCCTAA		
*C. psittaci* PCR	16S rRNA	C.p.16S 45F	TGGATGAGGCATGCAAGTCG	276	This study
		C.p.16S 320R	TGGCGGTCAATCTCTCAATC		
		C.p.16S 1172F	GGGTTAACCAGGAGGAAGGC	199	This study
		C.p.16S 1370R	AGCTGACACGCCATTACTAGC		
MLST PCR	*enoA*	YPenoA3	CCTATGATGAATCTCATTAATGG	428	(*22*); this study†
		YPenoA4	CCCAACCATCAAAATCTTCTTCCG		
	*fumC*	YPfumC1	GGGCTCCTGAGGTTATGCC	649	
		YPfumC2	CGCAAATAATGAATCACCTTATC		
	*gatA*	YPgatA3	GCCTTAGAGTTAAGAAATGCCG	509	
		YPgatA4	CCCCCTGTATCGGAACCTAACGC		
	*gidA*	YPgidA1	GCTTATTAGAGAGCTGTCCTGGC	693	
		YPgidA2	CGCGTTTTCTAACCCACGG		
	*hemN*	YPhemN1	GGATCCATTTCGGAGGAGGC	398	
		hemN-R2†	TAAGCGGTCAGGCCGCATGTG		
		hemN-F2†	GTCAAAGTCATGAAGAGTCAC	505	
		YPhemN2	CCTGAAAGGATTTTCTCATGG		
	*hflX*	YPhflX3	GAGATTTTTGCTAATCGAGCG	530	
		YPhflX4	GTAAAACATCTTCATGTAACGC		
	*oppA*	YPoppA3	ATGCGCAAGATATCAATGGG	500–610	
		YPoppA4	GGCAAGGTTTGGTGTAACTCGC		
PCR	*ompA*	CPsittGenoFor	GCTACGGGTTCCGCTCT	FO-01: 625; YO-02: 631; NO-03: 622	(*23*); this study†
		Cp ompA R3 †	CAATYTTAGGATTAGATTGAGC		
		Cp ompA F3 †	TGGGATCGCTYCGAYATTTTC	FO-01: 495; YO-02: 508; NO-03: 498	
		Cp ompA R4 †	TGCTCTTGACCAGTTTACGCC		
		Cp ompA F4 †	TATGGGAATGTGGTTGTGCAA	FO-01: 452; YO-02: 452; NO-03: 451	
		CPsittGenoRev	TTTGTTGATYTGAATCGAAGC		

*MLST, multilocus sequence typing.

We concatenated nucleotide sequences of 7 housekeeping genes for each strain among these 3 cases (FO-01, YO-02, and NO-03) and other strains (6BC, Mat116, and NJ1; GenBank accession nos. CP002586, CP002744, and CP003798), aligned them using MAFFT version 7 (*25*), and extracted single-nucleotide polymorphisms (SNPs) from the alignments using SNP-sites (*26*), then calculated the pairwise SNP distances using SNP-dists version 1.2.0 (https://github.com/tseemann/snp-dists). We visualized the resulting SNP distance matrix as a heatmap using the tidyverse version 2.0.0 and pheatmap version 1.0.13 packages in R version 4.5.1 (The R Project for Statistical Computing, https://www.r-project.org).

### Phylogenetic Analysis of the *ompA* Gene and OmpA Protein

We performed PCR of the *ompA* gene using placental DNA with the primers (Table 1). The *ompA* gene could not be amplified with the previously reported primer sets CPsittGenoFor and CPsittGenoRev; therefore, we designed new primers and obtained overlapping sequences. We performed both nucleotide and amino acid sequence analyses using sequences obtained from BLAST searches. We aligned nucleotide sequences with the representative *ompA* genotype sequence (*27*), converted nucleotide sequences to amino acid sequences, aligned them using MAFFT version 7 (*25*), trimmed for alignment optimization using trimAl and ClipKIT (*28*,*29*), and phylogenetically analyzed them with IQ-TREE version 3 (T.K.F. Wong et al., unpub. data, https://ecoevorxiv.org/repository/view/8916), including visualization using iTOL versin 7 (*30*).

### Quantitative Analyses

We performed qPCR targeting the 16S rRNA gene sequence of Chlamydiales using the primers CPSI_F and CPSI_R under previously described conditions. We performed relative quantification (RQ) using the 2^−ΔΔCt^ method, normalizing chlamydial DNA levels to β actin as internal control and calculating the relative fold change in the placenta and spleen compared with the lungs.

### Hematoxylin and Eosin and Immunofluorescence Staining

We examined hematoxylin and eosin–stained tissue sections under an Eclipse Ti microscope equipped with a DS-Fi3 camera (both Nikon, https://www.nikon.com). Using serial sections, we performed antigen retrieval with Dako Target Retrieval Solution, Citrate pH 6 (Agilent Technologies, https://www.agilent.com), and blocked the sections with 5% bovine serum albumin for 1 hour at room temperature. We stained the slides with a rabbit polyclonal (1:1,000) antibody raised against *C. psittaci* BC6 strain diluted with 1% bovine serum albumin (*10*). We used the anti-rabbit Alexa Fluor 488 (Thermo Fisher Scientific) as a secondary antibody and stained the nucleus with 4’,6-diamidino-2-phenylindole (Roche, https://www.roche.com). For immunofluorescence microscopy, we used a GFP/DAPI (green fluorescent protein/4′,6-diamidino-2-phenylindole) filter set (Chroma Technology Corp., https://www.chroma.com).

### Statistical Analyses

We analyzed data using JMP 18 statistical analysis software (JMP Statistical Discovery LLC, https://www.jmp.com) or Igor Pro 9.05 software (WaveMetrics, https://www.wavemetrics.com). We evaluated significant differences in the RQ of qPCR among tissues using 1-way analysis of variance, followed by pairwise comparison using Tukey–Kramer’s honest significant difference test. We considered a p value of <0.05 to be statistically significant.

### Nucleotide Sequence Accession Numbers and pubMLST Identification Numbers

The nucleotide sequences obtained in this study have been deposited in DDBJ/EMBL/GenBank under accession numbers LC900811 and LC900812 (FO-01), LC900813 and LC900814 (YO-02), and LC900815 and LC900816 (NO-03) for the partial 16S rRNA gene; LC921559–65 (FO-01), LC888489–95 (YO-02), and LC888482–8 (NO-03) for housekeeping genes using for MLST analysis; and LC486816 (FO-01, protein identification [ID] BBL33230), LC923014 (YO-02), and LC923015 (NO-03) for the partial *ompA* gene. The pubMLST isolate ID numbers are 4431, 5398, and 5399.

### Ethics Approval

Autopsy procedures were conducted with the informed consent of the families. This study was approved by the institutional review board and ethics committee of the Osaka Women’s and Children’s Hospital (no. 999, 999-2).

## Results

### Clinical Characteristics and Diagnosis of *C. psittaci*

The 3 cases FO-01, YO-02, and NO-03 occurred in different coastal cities in Japan in 2017, 2022, and 2024. In the case of FO-01, a 26-year-old pregnant woman demonstrated fever at 23 weeks and 4 days of gestation; vomiting began at 23 weeks and 6 days. In the case of YO-02, a 28-year-old pregnant woman demonstrated fever at 25 weeks and 5 days of gestation, and muscle pain also began at 26 weeks and 0 days. In the case of NO-03, a 32-year-old pregnant woman showed fever at 37 weeks and 2 days of gestation. The intervals from onset to death were 4 days in the case of FO-01, 4 days in the case of YO-02, and only 2 days in the case of NO-03. All 3 patients had fever and nonspecific symptoms, without respiratory complaints such as cough or sore throat (Table 2). At the initial visit, only mild elevations in leukocyte count and C-reactive protein were noted. Subsequently, septic shock developed in all 3 women. Laboratory tests revealed coagulation abnormalities, hepatic dysfunction, and renal impairment. Intrauterine fetal death was confirmed, and early termination was considered. However, because of the maternal poor systemic condition and coagulopathy, cesarean section could not be performed. Maternal death occurred before delivery of the fetus and placenta. On autopsy, although no infectious changes were observed in the lung, hemophagocytosis was noted in the spleen, consistent with hemophagocytic syndrome in all 3 cases.

**Table 2:** Clinical findings in 7 cases of gestational psittacosis reported in Japan during 2000–2024*

Case identification	Year	Maternal age	Gestational age at onset	Parity	Symptoms	Antibiotics administered	Maternal death	Fetal death	Reference
1	2000	Not reported	35 wks 3 d	Not reported	Fever, cough	CLR, MNO	N	N	(*31*)
2 (FO-01)	2017	26	23 wks 4 d	0	Fever, vomiting	None	Y	Y	(*17*,*18*); this study
3	2017	31	17 wks 1 d	0	Fever, malaise, headache, muscle pain	MEM	Y	Y	(*5*)
4	2021	31	15 wks 3 d	3	Fever, cough, headache	MEM, CSL, MTR, AZM	N	Y	(*32*)
5	2022	24	25 wks 2 d	0	Fever, malaise	TCY	N	N	(*33*)
6 (YO-02)	2022	28	25 wks 5 d	1	Fever, muscle pain	MEM	Y	Y	(*19**,**20*); this study
7 (NO-03)	2024	32	37 wks 2 d	2	Fever	Not reported	Y	Y	This study

*AZM, azithromycin; CLR, clarithromycin; CSL, cefoperazone/sulbactam; MEM, meropenem; MNO, minocycline; MTR, metronidazole; TCY, tetracycline.

In cases FO-01, YO-02, and NO-03, real-time PCR with primers CPSI_F and CPSI_R yielded positive results across all tissues. Pairwise comparison of the partial 16S rRNA gene sequences showed high similarity among the 3 isolates; percentage identities were 100.0% (FO-01-YO-02) and 99.8% (FO-01-NO-03 and YO-02-NO-03) as determined by BLAST analysis. Isolates LC900811 and LC900812 (FO-01) showed a 99.64% and 100% similarity to the 16S rRNA gene sequence of multiple *C. psittaci* strains, LC900813 and LC900814 (YO-02) showed a 99.64% and 100% similarity, and LC900815 and LC900816 (NO-03) showed a 100% and 100% similarity. On the basis of those results, *C. psittaci* infection was diagnosed in the patients.

### MLST Analyses

**Figure 1:**
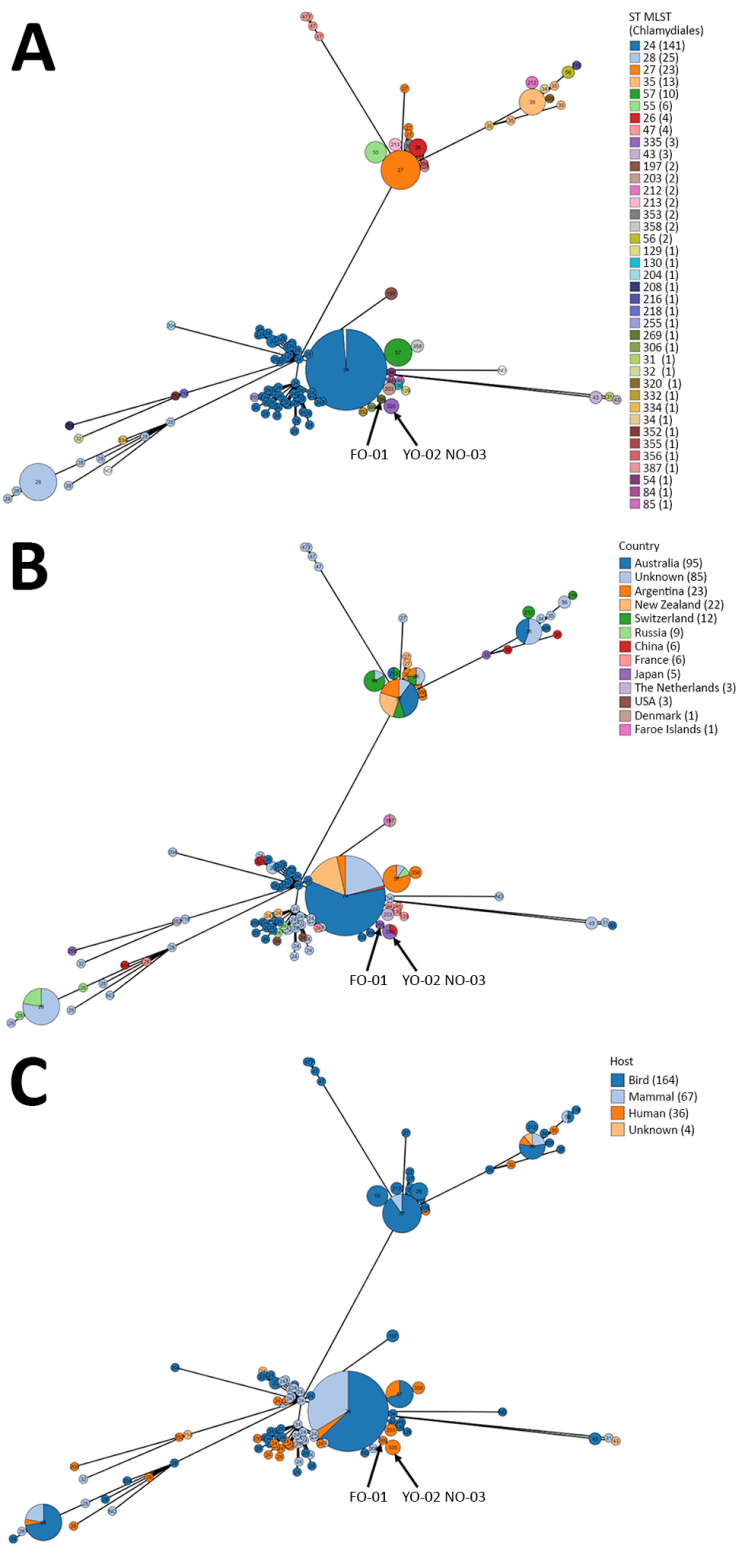
GrapeTree view showing MLST phylogenetic relationship among *Chlamydia psittaci* strains in study of 3 fatal gestational psittacosis cases (FO-01, YO-02, and NO-03) caused by *C. psittaci* strains belonging to closely related MLST lineages, Japan, 2017–2024. A) ST; B) country; C) source host. Strains include ST269/ST335. Numbers in parentheses indicate number of isolates. The MLST alleles are concatenated, and the ST is determined using the Chlamydiales database hosted at https://pubmlst.org/chlamydiales. MLST, multilocus sequence typing; ST, sequence type.

MLST analyses revealed that FO-01 belonged to ST269 and YO-02 and NO-03 belonged to ST335 (Table 3). According to the MLST phylogenetic analysis, ST335 and ST269 were closely related; ST335 was considered a derived ST from ST269, STs of which had not been previously reported in Japan or in animals. ST335 and ST269 showed c. 414C>T (p. Gly138 = , synonymous) and c. 515C>T (p. Pro172Leu, nonsynonymous) in *gidA* and c. 558G>A (p. Gly186 = , synonymous) in *hflX*. Phylogenetically, ST269/ST335 are distinct from the lineages, including ST208, which was previously reported in Japan and caused outbreaks in animals and humans at animal exhibition facilities, and ST35, which has been detected in asymptomatic feral parrots introduced to regions outside their native range (Figure 1). To date, ST269/ST335 occurrence has been limited to human patients; ST335 was isolated from bronchoalveolar lavage fluid of a male patient with pneumonia in China, then in the patients with gestational psittacosis in this study (*34*).

**Table 3:** Sequence type and allelic profiles of three cases of gestational psittacosis complicated by maternal and fetal death in study of 3 fatal gestational psittacosis cases caused by *C. psittaci* strains belonging to closely related MLST lineages, Japan, 2017–2024*

Case ID	Year	GenBank accession no.	PubMLST ID	Sequence type	*enoA*	*hemN*	*fumC*	*gatA*	*gidA*	*hflX*	*oppA*
1 (FO-01)	2017	LC921559–65	4431	269	13	9	78	85	92	90	78
2 (YO-02)	2022	LC888489–95	5398	335	13	9	78	85	112	108	78
3 (NO-03)	2024	LC888482–8	5399	335	13	9	78	85	112	108	78

*ID, identification.

**Figure 2:**
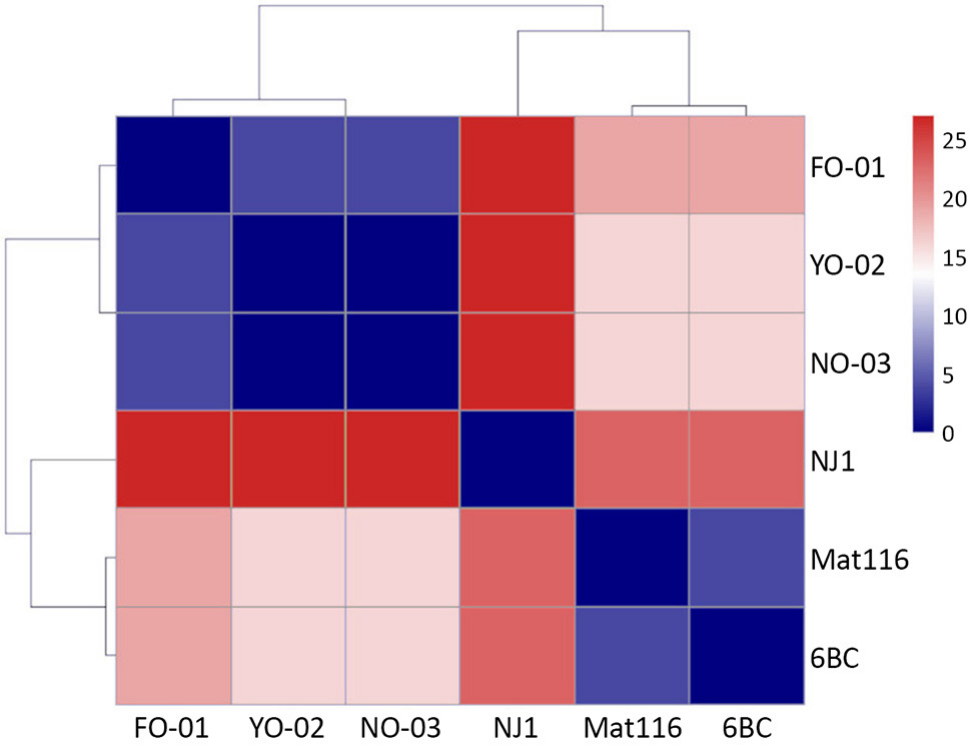
Heatmap of single-nucleotide polymorphism distances of housekeeping genes among 3 cases of gestational psittacosis (FO-01, YO-02, and NO-03) and other *Chlamydia psittaci* strains in study of fatal gestational psittacosis cases caused by *C. psittaci* strains belonging to closely related multilocus sequence typing lineages, Japan, 2017–2024.

The heatmap based on the SNP distances of 7 housekeeping genes also revealed that FO-01, YO-02, and NO-03 were closely related: there were 0 SNPs between YO-02 and NO-03, and FO-01 differed by 4 SNPs from both YO-02 and NO-03. Not only 6BC and NJ1 but also Mat 116, which was isolated in Japan, differed by 16–27 SNPs from FO-01, YO-02, and NO-03 (Figure 2).

### Phylogenetic Analysis Using the *ompA* Gene and OmpA Protein

**Figure 3:**
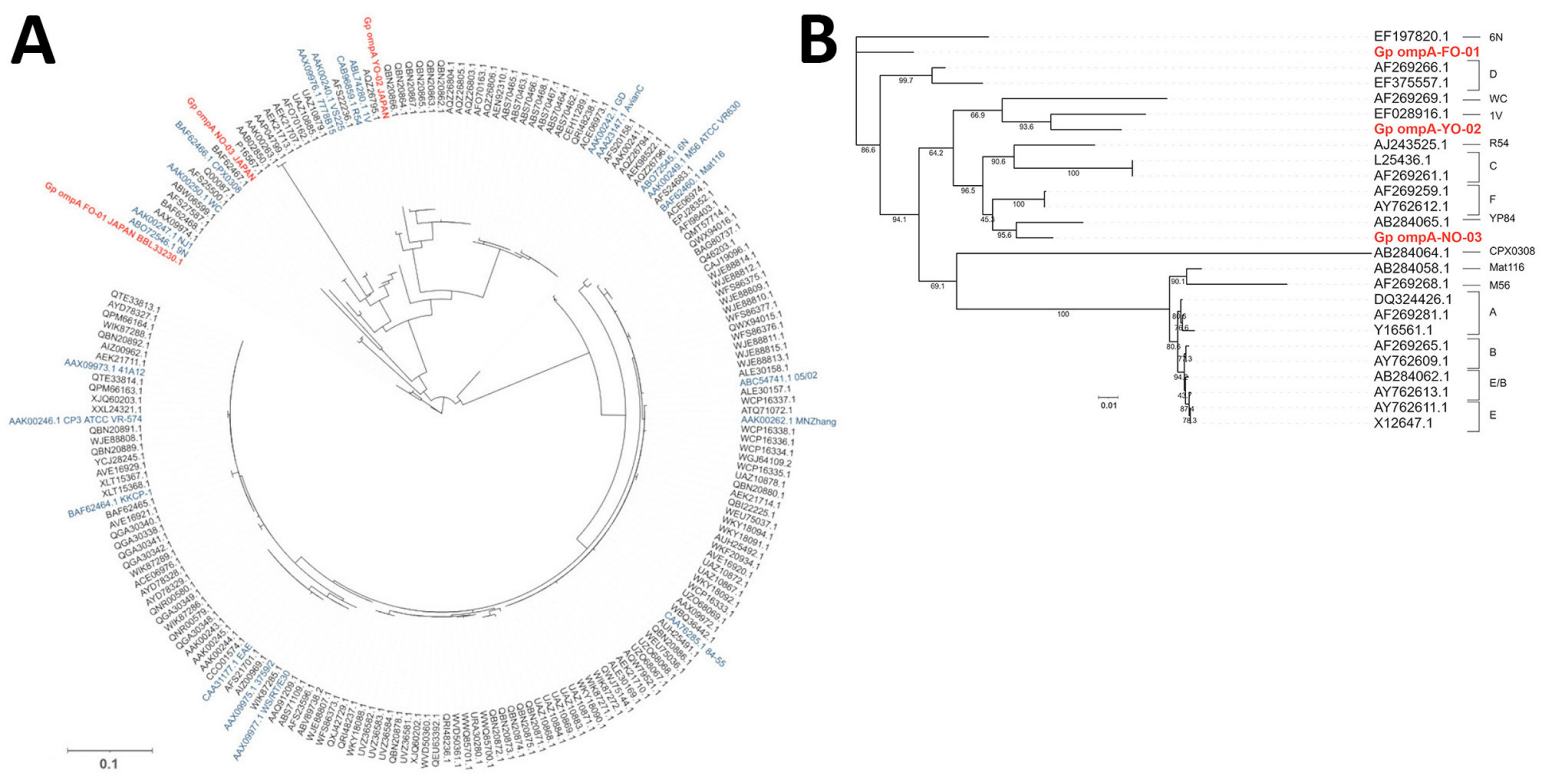
Phylogenetic analysis of the OmpA protein and *ompA* gene of *Chlamydia psittaci* in study of 3 fatal gestational psittacosis cases caused by *C. psittaci* strains belonging to closely related multilocus sequence typing lineages, Japan, 2017–2024*.* Red text indicates gestational psittacosis cases from in this study (FO-01, YO-02, and NO-03). A) Circular phylogenetic tree of the OmpA protein of 200 strains. Blue indicates representative *C. psittaci* strains. B) Phylogenetic tree of the *ompA* gene of the 3 strains from this study and representative *C. psittaci* genotypes. Scale bar indicates nucleotide or amino acid substitutions per site.

Phylogenetic analyses of the OmpA protein revealed that FO-01, YO-02, and NO-03 belonged to a different cluster, and sequence variations existed in the hypervariable region (Figure 3, panel A). FO-01 corresponded to protein ID BBL33230 and was the closest to genotype D. YO-02 was clustered in genotype 1V, and NO-03 was clustered in genotype YP84, which was isolated from a king parakeet in Japan (Figure 3, panel B).

### Chlamydial DNA Levels in the Placenta

**Figure 4:**
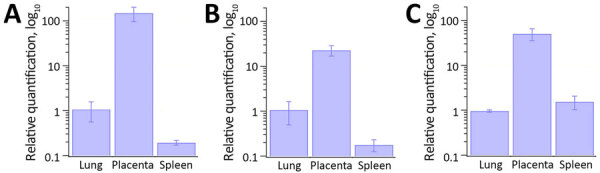
Relative quantification of chlamydial DNA from the maternal lungs, placenta, and spleen in study of 3 fatal gestational psittacosis cases caused by *C. psittaci* strains belonging to closely related multilocus sequence typing lineages, Japan, 2017–2024. Quantitative real-time PCR was performed targeting the 16S rRNA gene of the order Chlamydiales (n = 3). Relative quantification was performed using the 2^−ΔΔCt^ method, normalizing chlamydial DNA levels to β actin as internal control and calculating the relative fold change in the placenta and spleen compared with the lungs. A) Case FO-01; B) case YO-02; C) case NO-03.

In all 3 patients, qPCR indicated higher amounts of chlamydial DNA in the placenta than in the lungs and spleen (Figure 4). One-way analysis of variance revealed a significant difference in the RQ values of chlamydial DNA among tissues from the 3 female patients (case 1g F (2,6) = 23.8, p<0.01; case 2, F (2,6) = 41.7, p<0.001; case 3, F (2,6) = 32.0, p<0.001). Posthoc analysis using Tukey-Kramer’s honest significant difference test indicated significant differences of the placenta with the spleen and lungs (p<0.01), with ≈20- to 100-fold changes.

### *C. psittaci* Infection in Placenta

**Figure 5:**
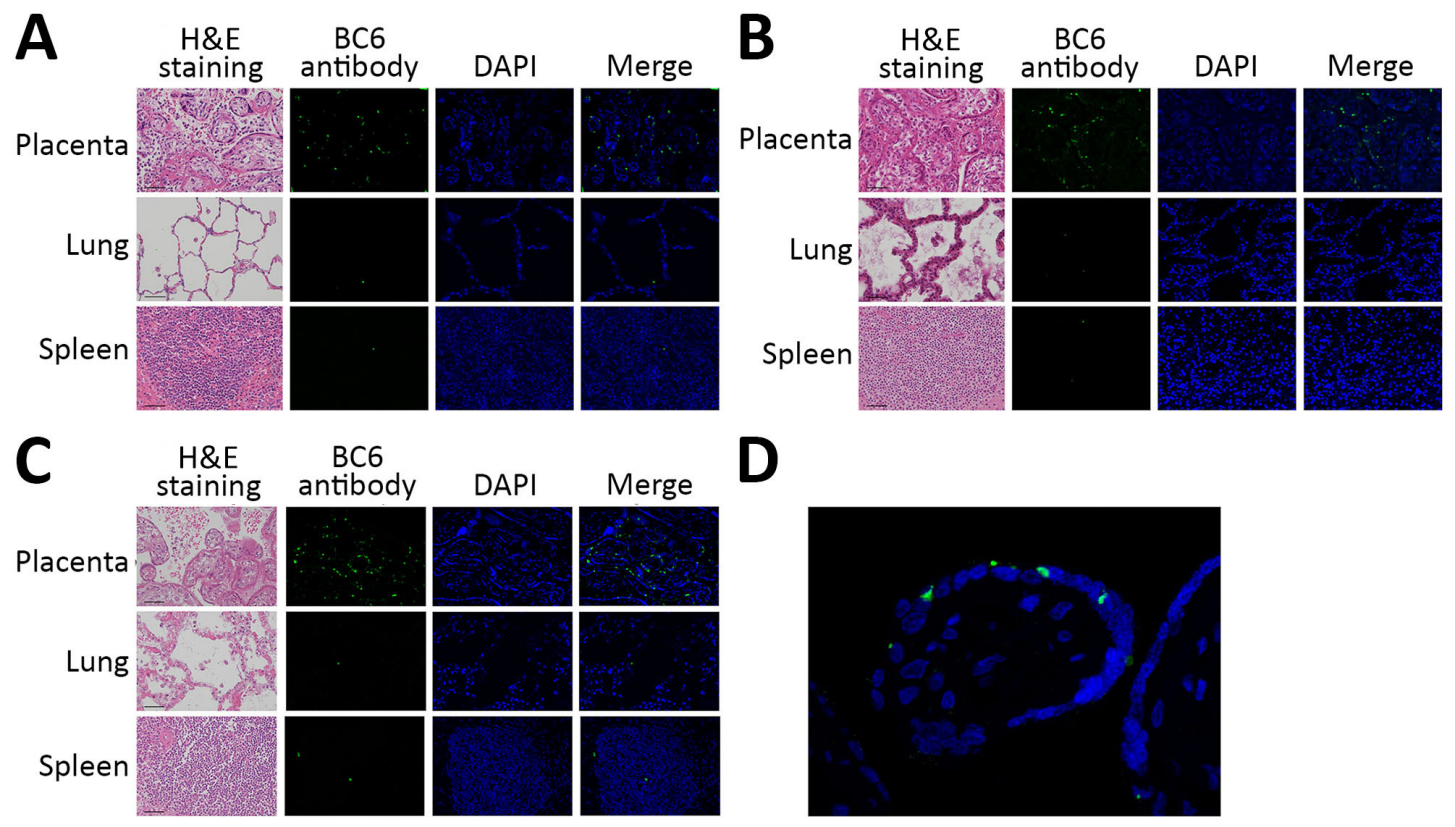
Histologic findings from 3 fatal gestational psittacosis cases caused by *C. psittaci* strains belonging to closely related multilocus sequence typing lineages, Japan, 2017–2024. A–C) Hematoxylin and eosin staining (scale bar = 50 µm; original magnification ×20) and immunofluorescence microscopy of the placenta, lung, and spleen are shown for case FO-01 (A), case YO-02 (B), and case NO-03 (C). Specific fluorescence observed via immunofluorescence using *C. psittaci* BC6 rabbit antibody staining. Nuclei were stained with DAPI. Merge column indicates BC6 antibody staining and DAPI. Original magnification ×20. D) High-magnification view (original magnification ×60) of the villi. DAPI, 4′,6-diamidino-2-phenylindole; H&E, hematoxylin and eosin.

Hematoxylin and eosin staining showed marked intervillositis in the placenta, with pronounced inflammatory changes compared with the changes seen in the lung and spleen. Immunofluorescence staining revealed higher signals in placenta compared with lungs and spleen in all 3 cases. *C. psittaci* was markedly observed, especially in the syncytiotrophoblast cells (Figure 5).

## Discussion

In Japan, there have been 7 cases of gestational psittacosis, resulting in a total of 4 maternal deaths (mortality rate 57.1%) and 5 fetal deaths (mortality rate 71.4%) (Table 2) (*5*,*17*–*20*,*31*,*32*,*33*). Of note, the route of infection remained unknown in all reported cases, except in 1 patient (case ID 1 in Table 2) who kept a parrot. Reports on patients with gestational psittacosis in various regions of Japan have been sporadic, making epidemiologic analysis difficult and control of this infectious disease challenging. After the announcement of the new classification of *C. psittaci* and *C. abortus* in 1999 (*35*), 5 cases of gestational psittacosis were reported in China during 2020–2025, 1 case in the United States in 2018, and 3 cases in Europe during 2006–2022. All cases in China had a history of contact with birds (parrots, pigeons, and poultry), whereas all cases in Europe had a history of contact with sheep (1 case also had contact with goats). The case in the United States had contact only with a pet cat as the animal source, and the clear source of infection was unknown (*36*–*43*; X. Wu et al., unpub. data, https://www.researchsquare.com/article/rs-53548/v1).

In Japan, all 3 surviving mothers had been administered antimicrobial agents, such as macrolides and tetracycline. However, for the 4 mothers who died, their conditions rapidly became severe after hospitalization, and no treatment was effective. Pneumonia was diagnosed in only 1 patient, and the other patients had no signs of respiratory infectious disease.

The fatal gestational psittacosis cases we describe belonged to closely related ST269/ST335 lineages, as indicated by MLST analysis. The phylogenetic analysis of the *ompA* gene revealed differences in the hypervariable region, grouping them into distinct clusters. Because the cases (FO-01, YO-02, and NO-03) occurred in geographically separate locations in Japan, they are considered to be independent cases of infection rather than an outbreak originating from a single source. MLST is a phylogenetic analysis using highly conserved housekeeping genes; the fact that the strains were identified as closely related in this analysis suggests that they might share a common phylogenetic background.

In this study, the higher bacterial loads in placentas than in maternal lungs and spleens indicated a pronounced proliferative potential of *C. psittaci* in the human placenta. The association of ST269/ST335 lineages with *C. psittaci* proliferation in human placenta remains unclear. Epidemiologically, in Japan, ST269/ST335 lineages have not been previously reported in humans and animals. Elucidating the sources of infection of those lineages remains crucial for effective prevention. Mat116 lineage was reported to circulate between human patients and wild birds (*44*). However, no obvious psittacosis patients were among the family members and medical personnel who had contact with these gestational psittacosis patients. Considering that human-to-human transmission has not been ruled out and the possibility that this *C. psittaci* strain is highly pathogenic to pregnant women, more detailed epidemiologic information on humans and animals is needed.

Diagnosis of *C. psittaci* infection is often challenging because of its nonspecific symptoms, such as fever, as seen in the patients we describe. The rarity of the disease makes it challenging to consider in the differential diagnosis, and the diagnostic tests, such as isolation of *C. psittaci*, serology, and detection of *C. psittaci* DNA, are not routinely performed in most hospitals (*1*). Undiagnosed *C. psittaci* infection could account for some cases of sudden, unexplained maternal death, suggesting that the actual burden of gestational psittacosis is likely underestimated worldwide.

The cases we report, and other reports on gestational psittacosis, have noted extensive intervillous inflammation of the placenta on pathological examination (*17*–*20*,*32*). Acute intervillositis represents hematogenous spread of organisms from the mother to the fetus through the placenta, and *C. psittaci* is thought to reach the placenta through hematogenous spread. The presence of severe symptoms in pregnant women could be explained several ways. First, as pregnancy progresses through the second and third trimesters, the immune system shifts toward a tolerogenic state to support the developing semiallogenic fetus, uterine dendric cells and natural killer cells produce the regulatory interleukin 10, systemic Treg cell activity increases, and cytotoxic leukocyte activity decreases. Simultaneously, overall Th immunity shifts toward a Th2 profile, mediated in part by differentiation of naive T cells to Th2 cells and accumulation of Th2 cells in the uterus (*45*). That immunologic environment, characterized by a shift from Th1 to Th2 dominance, potentially favors the proliferation of intracellular bacteria, such as *C. psittaci*, which rely on evading Th1-type immune response. Second, during pregnancy, plasma volume at term can increase by nearly 1 L, 90% of which is used in the placenta (*46*). Those hemodynamic changes during pregnancy might enable the spread of *C. psittaci* to the placenta. Compared with FO-01 and YO-02 in this study, NO-03 showed more rapid progression of the initial symptoms to maternal and fetal death at 37 weeks of gestation. That difference might reflect the high proliferative capacity of *C. psittaci* within the placenta, where bacterial load appears to correlate with placental weight (*47*). Fetal death in those 3 cases was considered to be associated with maternal systemic deterioration and placental dysfunction.

The first limitation of our study is that only fixed tissue samples were available, which precluded pathogen isolation and evaluation of virulence in animal models. Because DNA was extracted from FFPE tissue, only short DNA fragments could be amplified; we could not yet obtain the entire genome sequence. However, we successfully amplified and obtained the sequences required for MLST and *ompA* analyses, enabling reliable strain characterization. In addition, genetic data on *C. psittaci* in Japan remain scarce, limiting the generalizability of our findings.

In conclusion, our study identified ST335/ST269 as potential highly virulent lineages in gestational psittacosis. Continued surveillance, improved diagnostic approaches, and further research on transmission pathways and pathogenicity are needed. Our findings provide a basis for risk stratification and targeted surveillance, supporting earlier clinical recognition and prevention of severe outcomes in pregnant women.
